# Dynamic Performance Evaluation of Bidirectional Bridgeless Interleaved Totem-Pole Power Factor Correction Boost Converter

**DOI:** 10.3390/mi16020223

**Published:** 2025-02-16

**Authors:** Hsien-Chie Cheng, Wen-You Jhu, Yu-Cheng Liu, Da-Wei Zheng, Yan-Cheng Liu, Tao-Chih Chang

**Affiliations:** 1 Department of Aerospace and Systems Engineering, Feng Chia University, Taichung 407, Taiwan; 2 Ph.D Program of Mechanical and Aeronautical Engineering, Feng Chia University, Taichung 407, Taiwan; p1136209@o365.fcu.edu.tw; 3 Electronic & Optoelectronic System Research Laboratories, Industrial Technology Research Institute, Hsinchu 31040, Taiwan; louisliu@itri.org.tw (Y.-C.L.); dawei_zheng@itri.org.tw (D.-W.Z.); rainliu@itri.org.tw (Y.-C.L.); taochih@itri.org.tw (T.-C.C.)

**Keywords:** on-board charger, power factor correction, totem-pole interleaved boost converter, dynamic characteristics, power efficiency, SiC power module

## Abstract

This study aims to conduct an assessment of the dynamic characteristics of a proposed 6.6 kW bidirectional bridgeless three-leg interleaved totem-pole power factor correction (PFC) boost converter developed for the front-end stage of electric vehicle onboard charger applications during load cycles. This proposed PFC boost converter integrates the self-developed silicon carbide (SiC) power MOSFET modules for achieving high efficiency and high power density. To assess the switching transient behavior, power loss, and efficiency of the SiC MOSFET power modules, a fully integrated electromagnetic-circuit coupled simulation (ECCS) model that incorporates an electromagnetic model, an equivalent circuit model, and an SiC MOSFET characterization model are used. In this simulation model, the impact of parasitic effects on the system’s performance is considered. The accuracy of the ECCS model is confirmed through comparing the calculated results with the experimental data obtained through the double pulse test and the closed-loop converter operation. Furthermore, a comparative study between the interleaved and non-interleaved topologies is also performed in terms of power loss and efficiency. Additionally, the performance of the SiC MOSFET-based PFC boost converter is further compared with that of the silicon (Si) insulated gate bipolar transistor (IGBT)-based one. Finally, a parametric analysis is carried out to explore the impact of several operating conditions on the power loss of the proposed totem-pole PFC boost converter.

## 1. Introduction

With the rapid growth of the electric vehicle (EV) market, the demand for onboard chargers (OBCs) is experiencing a fast upward trend [1]. As the core component of the EV charging system, the OBC is primarily responsible for converting the alternating current (AC) supplied by the external power grid into the direct current (DC) needed for charging the vehicle’s high-voltage battery pack, enabling efficient energy conversion from the grid-to-vehicle (G2V) [2]. The performance of the OBC not only directly impacts the charging efficiency and charging time of the EVs but also plays a critical role in overall energy utilization and system reliability. With the growing market demand for designs with fast charging, high efficiency, and high power density, innovations and developments in OBC technology have become a crucial research direction in the EV industry. A typical OBC system consists of two main functional modules: the front-end AC/DC converter and the back-end isolated DC/DC converter, each responsible for different essential functions [3,4]. The front-end AC/DC converter, besides converting the AC power from the grid into stable DC power, is in charge of controlling the input current to remain in phase with the input voltage through the power factor correction (PFC) technology. This not only effectively improves the power factor (PF) but also significantly reduces harmonic distortion on the input side, meeting the requirements for electromagnetic compatibility (EMC) standards. In addition, the front-end AC/DC converter must maintain stable output performance under varying input voltage and load conditions, which imposes stringent requirements on the system’s design and control strategy. On the other hand, the back-end isolated DC/DC converter allows the DC voltage to stabilize, after being converted by the front-end converter, within the range suitable for battery charging, and provides electrical isolation to ensure the safety of the system’s operation. This module not only accommodates diverse battery charging requirements but also enhances the overall system’s stability and reliability through its isolation design. In the overall OBC system architecture, the role of the front-end AC/DC converter is particularly vital, as it directly impacts the stability of the output DC voltage, power conversion efficiency, and the system’s energy consumption level. An efficient and stable front-end converter can lower energy losses during the charging process, and also minimize system heating, thereby improving the system’s reliability and lifetime. Meanwhile, an optimized front-end design helps to lessen the size and weight of the OBC system, meeting the EV’s demand for high power density and lightweight solutions. Accordingly, in-depth research and performance improvement of the front-end AC/DC converter have become a key direction for accomplishing high-efficiency OBC systems, and are one of the core challenges for the future development of the OBC technology.

The most common topology for PFC is the traditional bridge rectifier. The traditional bridge rectifier configuration uses a diode bridge to convert the AC voltage into pulsating DC voltage, which is then stabilized into a steady DC output voltage through a boost converter. This configuration confers several advantages, such as simple design, low cost, and slower control loop response, making it widely used in various applications with low power requirements. However, the traditional bridge rectifier also has several drawbacks, including high diode conduction loss, low PF, significant harmonic distortion, and lack of support for bidirectional power flow. These limitations make it difficult to meet the demand of modern high-performance power electronic systems [5]. To tackle the limitations of the traditional bridge rectifier configuration, bridgeless PFC configuration was proposed (see, e.g., [6]). Bridgeless configuration eliminates the traditional diode bridge, and instead uses active switching components for rectification, which leads to a significant reduction of conduction loss and better system efficiency. This configuration not only lessens the number of components and power loss but also supports bidirectional power operation, further fulfilling the demands for high efficiency and high-power density in modern applications, such as EVs [7]. Common bridgeless PFC configurations include the interleaved boost PFC [8] and the totem-pole PFC [9,10,11,12]. Among them, the totem-pole PFC configuration is gradually becoming the focus of research and application due to its simple structure and superior performance. Additionally, it can further optimize the performance of the bridgeless PFC boost converters, with the main advantage being the elimination of the traditional diode bridge [9,13]. In addition, it enables bidirectional operation, which is particularly crucial for applications such as vehicle-to-grid (V2G) [14]. However, this architecture faces mode transition issues at the zero-crossing point of the AC voltage [11,15]. The intrinsic body diodes in the traditional silicon (Si) MOSFET power devices possess poor reverse recovery characteristics, which leads to huge reverse recovery charge and hard switching phenomena, making it prone to increased power losses and electromagnetic interference (EMI) issues [12,16,17,18]. To address this problem, third-generation semiconductor materials, including silicon carbide (SiC) and gallium nitride (GaN), have been introduced to the totem-pole PFC design [19]. These materials have inherent characteristics, such as high switching speed, low conduction losses, and low reverse recovery charge, which not only mitigate the hard switching issues but also enable operation under high-voltage and high-temperature conditions [20,21,22,23,24,25]. However, as power and switching frequency increase, the impact of parasitic effects [26,27,28,29,30] becomes more pronounced, which leads to additional power losses, further causing increased device junction temperature and thermal management challenges. Temperature is one of the most critical and challenging issues in power electronics applications [31,32,33].

Parasitic parameters, including inductance and capacitance, are critically affected by the module design and circuit layout. Parasitic inductance can cause voltage spikes and oscillations during switching transients, which not only elevate switching losses but may also bring about EMI issues. Parasitic capacitance, on the other hand, has a critical impact on the voltage and current conversion process, exacerbating energy loss during switching and subsequently influencing overall system loss. More importantly, parasitic effects tend to become more significant under high-frequency operation, resulting in decreased system efficiency and an increased burden on thermal management.

Therefore, accurately assessing the efficiency and power losses of OBC systems has been a central research topic for further electrical, thermal and reliability performance design and improvement. Recently, many studies have been conducted to evaluate the performance of various power modules for OBC systems through theoretical analysis and experimental characterization [10,12,13,14,30,34]. For example, Rong et al. [17] conducted modeling and simulation using MATLAB/Simulink to study hard-switching circuits, specifically the boost converter, and demonstrated that the boost circuit performed well in PFC circuit design. Dini and Saponara [30] conducted a detailed discussion of a bidirectional single-phase 7 kW OBC using both electrical and thermal models. They explored the impact of various operating conditions on the output voltage and the temperature oscillations during system operation. Su and Lu [34] proposed an interleaved totem-pole boost bridgeless rectifier and achieved zero-voltage switching by using SiC MOSFETs under low voltage input. Additionally, an 800 W experimental setup was constructed to verify the feasibility of the theoretical analysis. Their results showed that the interleaved totem-pole PFC could achieve a reduced conduction loss, attaining high efficiency and bidirectional operation.

Although there have been some studies in recent years on parasitic inductance, switching waveforms, system operation, and performance of PFC converters, comprehensive system exploration from the design to the operation of the OBC front-end totem-pole PFC converter remains limited. Therefore, this study attempts to investigate the power losses, PF, efficiency, and switching transients of a developed SiC MOSFET-based 6.6 kW bidirectional bridgeless three-leg interleaved totem-pole PFC boost converter for use in the front-end stage of OBCs for EV applications during load operation. To address the parasitic effect on the dynamic characteristics of the PFC boost converter, a fully integrated electromagnetic-circuit coupled simulation (ECCS) model is introduced. The calculated results are compared to the experimental data of the double pulse test (DPT) and the closed-loop converter operation. Furthermore, the converters with interleaved topology and non-interleaved topologies are compared with each other in terms of power losses and efficiency. Apart from that, the performance comparison between the SiC MOSFET-based and the Si insulated gate bipolar transistor (IGBT)-based interleaved totem-pole PFC boost converters is presented. Finally, a design guideline for improved power loss of the developed totem-pole PFC boost converter is sought through parametric analysis.

## 2. SiC MOSFET-Based Bidirectional Bridgeless Interleaved Totem-Pole PFC Boost Converter

**Figure 1:**
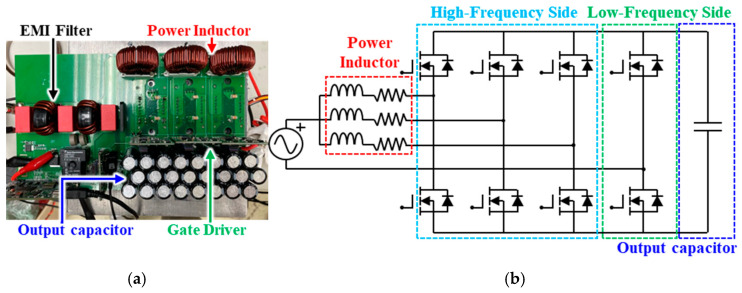
Bidirectional bridgeless three-leg interleaved totem-pole PFC boost converter: (**a**) Prototype; (**b**) circuit topology.

In this investigation, a bidirectional bridgeless interleaved totem-pole PFC boost converter is introduced, as shown in Figure 1a, with circuit topology illustrated in Figure 1b. The interleaved boost converter mainly consists of a three-leg system, high-frequency side power switches, and low-frequency side power switches. Each leg in the three-leg system is composed of a series-connected inductor and a resistor, and these inductors and resistors have an equivalent inductance and resistance. In view of the fact that SiC power MOSFETs feature an exceptionally low on-state resistance and SiC body diodes exhibit a relatively small reverse recovery charge, they are particularly well-suited for applications in bidirectional OBCs to achieve high efficiency and cost-effectiveness.

**Figure 2:**
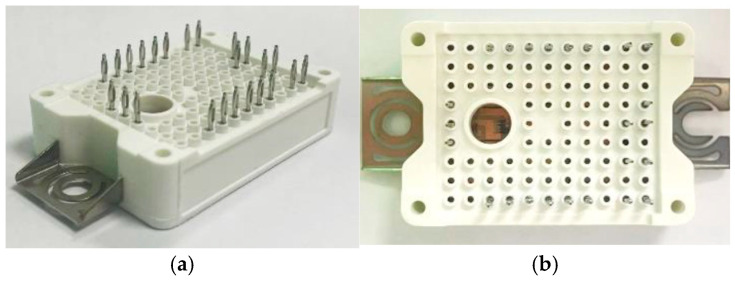
Developed 1700 V/100 A SiC MOSFET power module: (**a**) iso-view; (**b**) top view.

The high-frequency side, as clearly shown in Figure 1b, comprises three-connected, single-phase, half-bridge SiC MOSFET power modules made by Industrial Technology Research Institute (ITRI) (see, Figure 2), to form a three-phase topology. Each of these three-connected single-phase, half-bridge SiC MOSFET power modules consists of two series-connected power switches, one on the upper arm and the other on the lower arm, and each power switch encompasses two GeneSiC 1.7 kV/80 A SiC MOSFET power devices connected in parallel to increase the current rating. The power switches in each phase operate at a switching frequency of 100 kHz, and the phase angle between the power switches of these three phases differs by 120 degrees. The length, width and height of the power MOSFET device is 7.3 × 4.6 × 0.15 (mm). The SiC MOSFET device consists of a 38 aluminum (Al) pad where Al wires are connected to the surface of the Al pad. SAC305 lead-free solder is utilized for die bonding of the SiC MOSFET devices to a direct bonded copper (DBC) substrate comprising a top copper (Cu) circuit layer of 0.3 mm thick, an Al2O3 insulating layer of 0.32 mm thick, and a bottom Cu layer of 0.3 mm thick. In addition, these SiC MOSFET devices and Al wire bonds are protected by polyphenylene sulfide housing and silicone gel. On the other hand, the low-frequency side comprises four commercial Wolfspeed TO-247 SiC MOSFET power modules, which are used to constitute two series-connected power switches, serving, respectively, as the lower-arm switch and upper-arm switch. The power switches operate at the line frequency of 60 Hz. Each of these two power switches encompasses two parallel-connected 1.2 kV/60 A SiC MOSFET devices. These switches primarily act as synchronous rectification, causing negligible switching loss. Considering that the nominal voltage of most EV batteries is 400 V, the output voltage of this design is set to 400 V, with a rated power of 6.6 kW [35].

The converter design operates in continuous conduction mode (CCM) and implements three-leg interleaving. The application of multi-leg interleaving technology in totem-pole PFC boost converters demonstrates significant system advantages, particularly making them prone to offering greater practicality under demands for high efficiency and high power density. Through the three-leg interleaved design, the input current is evenly distributed across each leg for processing, and thus, the total current ripple and the current peaks can be substantially diminished. These characteristics not only alleviate the stress on the power inductors but also effectively enhance the system’s EMC performance. By uniformly distributing the input current across these three legs, the current stress on each of these power devices is reduced. It is crucial to note that reduced current stress would decrease the conduction and switching losses and so improve overall system efficiency. Additionally, the uniformly distributed current load helps to improve thermal distribution. An improved thermal distribution could potentially lower the risk of localized thermal concentration and thus heighten the reliability of the power system and the lifetime of the power components.

In the totem-pole PFC boost converter, the power inductor plays a crucial role in energy storage and current regulation, where it can significantly impact the system’s operational efficiency and power loss [36]. For example, the power inductors store the energy supplied by the AC voltage during the switching cycle and facilitate energy transfer and conversion during the ON and OFF states of the switching devices. Moreover, the power inductor could also effectively limit the rate of current change, smooth the input current waveform, and ensure the input current remains in phase with the AC voltage. In CCM, the power inductors help to maintain current continuity and lessen current peaks; as a result, the switching and conduction losses of power devices are lessened and the overall system’s efficiency is improved. Consequently, selecting an appropriate inductor is of critical importance. The inductance value is determined based on the input voltage, output voltage, and worst-case ripple conditions. The current converter design seeks to achieve a current ripple of less than 5%, which can be calculated as [9],

(1) $$I_{ripple}<5\%\times \frac{\sqrt{2}\times P_{out}}{V_{in}\times \eta }$$

where $P_{out}$ is the output power, η is the efficiency, and $V_{in}$ is the input voltage. The calculation of the current ripple can be divided into three regimes:

(2) $$I_{ripple}=\left[\frac{V_{in}}{L}-2\times \frac{\left(V_{out}-V_{in}\right)}{L}\right]\times D\times \frac{1}{f_{sw}},\;0<D\leq \frac{1}{3}$$

(3) $$I_{ripple}=\left[\frac{2\times V_{in}}{L}-\frac{\left(V_{out}-V_{in}\right)}{L}\right]\times \left(D-\frac{1}{3}\right)\times \frac{1}{f_{sw}},\;\frac{1}{3}\leq D<\frac{1}{3}$$

(4) $$I_{ripple}=\left[\frac{3\times V_{in}}{L}\right]\times \left(D-\frac{2}{3}\right)\times \frac{1}{f_{sw}},\;\frac{2}{3}\leq D<1$$

Under the worst-case conditions, the equation becomes:

(5) $$I_{ripple}=\frac{V_{out}}{12\times L\times f_{sw}}$$

where $V_{out}$ is the output voltage, D is the duty cycle, and $f_{sw}$ is the switching frequency. With the output power of 6.6 kW, and the root mean square (RMS) input voltage of 220 *V_rms_*, the RMS current *I_rms_* is found approximately 30 A, the corresponding inductance value is calculated to be around 222 µH. Hence, an inductor value of 270 µH is selected for this design. In the converter system, the primary function of the output capacitor is responsible for stabilizing the DC output voltage and minimizing the voltage ripple for stable operation and high power quality. Its value is primarily determined by the ripple magnitude, as given by [10],

(6) $$C_{out}=\frac{P_{out}}{\Delta V_{out}\times 2\pi \times f_{ac}\times V_{out}^{2}},$$

where $f_{ac}$ is the line frequency, and $\Delta V_{out}$ is the magnitude of the output voltage ripple. The current design aims for a ripple of less than 6.5%, leading to a calculated capacitance value of approximately 875 µF, and eventually, a capacitor value of 900 µF is used. The parameters for the developed interleaved totem-pole PFC boost converter are shown in Table 1.

## 3. Experiments

### 3.1. Double Pulse Test (DPT)

**Figure 3:**
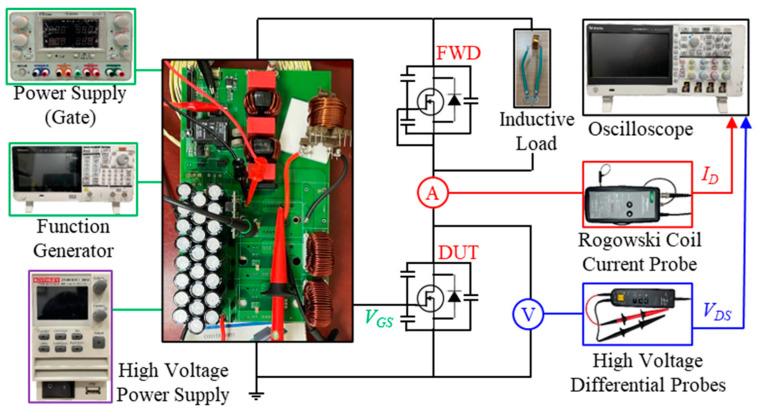
DPT experiment configuration.

DPT is a widely used approach to characterize the switching parameters and behavior of power devices. The turn-on and turn-off processes obtained through the DPT effectively evaluate the switching performance of the power devices under different operating conditions. It is thus applied to measure the switching characteristics of the SiC power MOSFETs used in the totem-pole PFC system. For the DPT experiment that follows the IEC 60747-8 international standard for testing [37], the measurement circuit is depicted in Figure 3. The test circuit adopts a half-bridge configuration, with one SiC MOSFET power switch acting as a freewheeling diode (FWD) for freewheeling, and the other serving as the device under test (DUT) for switching control. In the experiment, the gate and source of the FWD are shorted, while the gate and source of the DUT are connected to the control board. The gate drive circuit provides a gate drive voltage range of −4 V to 15 V for precise control of the power switches. The experimental setup includes a high-voltage power supply, a high-frequency oscilloscope, a high-voltage differential probe, a function generator, a Rogowski coil current probe, a load inductor, a gate driver board, and a power board. The DC bus voltage is set to 1200 V, with a rated current of 100 A, and the load inductance is set to 154 µH. The width of the first pulse is set to 15 µs to adjust the inductor current and safeguard it to reach the rated value. Based on the relationship between the inductor voltage and current, when the DUT turns on, the current in the load inductor gradually increases and reaches the rated current of 100 A during the first pulse period. Once the current attains the rated value, the gate control signal of the DUT is turned off, causing the current flowing through the load inductor to flow through the freewheeling diode. The load current remains stable, ensuring that when the DUT turns on again during the second pulse, the load current can be accurately restored to the rated value. During the test, the current probe is connected in series between the DC bus capacitor and the drain of the DUT to measure the drain current ($I_{d}$). The high-voltage differential probe is connected in parallel across the DUT to measure the drain-source voltage ($V_{ds}$). By recording the ON and OFF waveforms of the DUT, the switching time and characteristics can be accurately quantified.

### 3.2. Closed-Loop Totem-Pole PFC Converter Operation Experiment

**Figure 4:**
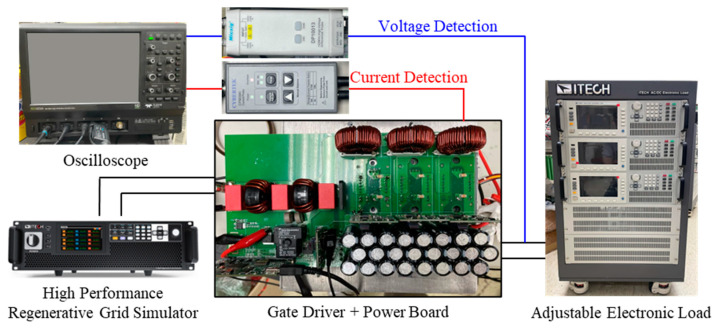
Closed-loop PFC converter operation experiment configuration.

The proposed interleaved totem-pole PFC boost converter is utilized for the front-end AC/DC conversion of OBCs. To characterize its actual output performance, an experiment is conducted on the closed-loop operation of the totem-pole PFC boost converter. The operational settings of the totem-pole PFC boost converter are displayed in Figure 4. The experimental setup includes an AC power supply, a power board, gate drivers, power modules, power inductors, an output capacitor, and an adjustable electronic load. The AC power supply provides a stable 220 *V_rms_*, 60 Hz AC input to simulate actual grid conditions and serves as the energy source for the system. The power board, as the central control unit of the entire system, integrates an EMI filter and a microcontroller unit (MCU). The EMI filter is responsible for suppressing high-frequency noise to ensure that the system meets EMC requirements. The MCU executes the closed-loop control strategy, including precise regulation of the input voltage and current, to achieve the PFC objectives. The power conversion unit of the system is composed of power modules and gate drivers. The power modules incorporate SiC MOSFET devices for high-frequency switching operations, in which a switching frequency of up to 100 kHz is supported to enable high-performance power conversion. The gate driver provides precise control signals, and thus facilitates the coordinated operation of the high-frequency and low-frequency switching. A 400 ns dead-time delay is configured to prevent cross-conduction between the high-side and low-side power switches, thereby safeguarding the system’s operational safety. In each leg of the power circuit, a 270 µH power inductor is used for energy storage and current ripple suppression [38], which confers smooth input current and maintains a high PF for the system. The DC bus utilizes an output capacitor bank comprising 24 capacitors, each rated at 350 V and 150 µF. Two capacitors are connected in series to form 12 parallel groups, which give sufficient energy storage capacity to stabilize the DC output voltage. The system’s output load is implemented using an adjustable electronic load, which simulates operating conditions under various load scenarios and evaluates the system’s performance at the rated output power of 6.6 kW.

## 4. Theoretical Model

### 4.1. Definition of Power Factor (PF)

In an AC power system, PF is a crucially important metric describing the efficiency of energy transfer within the system. Since the load in an AC power system typically consists of inductive, capacitive, and resistive components, a phase difference emerges between the input voltage and current. This phase difference brings about a discrepancy between the apparent power (*S*) and the real power (*p*), which represents the actual energy transfer. A low PF causes reduced utilization of the capacity of power transmission and distribution equipment, increased system losses, and even potential impacts on voltage stability. The mathematical definition of the PF is the ratio of real power to apparent power as:

(7) $$PF=\frac{P}{S}$$

Real power is the active portion of power absorbed by the load, which is directly converted into useful energy, such as heat, mechanical energy, or other forms of effective work. It is denoted as:

(8) $$P=V_{rms}\times I_{rms}\times \cos\theta ,$$

where cosθ denotes the displacement PF (DPF), which is the cosine of the phase angle θ between the fundamental components of the voltage and current. Apparent power is the product of the RMS values of voltage and current, reflecting the total power demand in the system, as,

(9) $$S=V_{rms}\times I_{rms}$$

The value of the PF ranges between 0 and 1. A value closer to 1 represents a higher energy transfer efficiency of the system. This indicates that the voltage and current waveforms are closer to ideal sine waves and are in phase, with a greater proportion of the apparent power being effectively converted into real power. In practical applications, the PF must also account for the harmonic components in the current. The RMS value of the current can be divided into the RMS value of the fundamental component ($I_{1,rms}$) and the RMS value of the distortion component ($I_{1,dis}$), with their relationship expressed as,

(10) $$I_{rms}^{2}=I_{1,rms}^{2}+I_{dis}^{2}.$$

The total harmonic distortion caused by harmonic distortion is defined as,

(11) $$\zeta =\frac{I_{dis}}{I_{rms}}\times 100\%.$$

Therefore, the PF can be further written as,

(12) $$PF=\frac{1}{\sqrt{1+\zeta^{2}}}\cdot DPF.$$

### 4.2. Power Loss Estimation

In the totem-pole PFC boost converter system, various power losses inevitably occur within the system during operation. These losses are primarily attributed to the switching and conduction losses of the SiC MOSFET devices, as well as the conduction and reverse recovery losses of the SiC body diodes [28]. When the upper arm power switch changes from the OFF state to the ON state, the charge stored in the parasitic capacitance of the body diode is quickly released, causing a reverse current during the transient period, resulting in the reverse recovery loss [39,40]. The conduction loss is the energy dissipated due to the current flowing through the internal channel of the device when it is in the ON state, as a result of the conduction resistance ($R_{ds\left(on\right)}$). The magnitude of the conduction loss can be calculated using the following formula:

(13) $$P_{Cond}=I_{rms}^{2}\times R_{ds(on)}$$

On the other hand, during the operation of the power MOSFETs, switching loss results from the simultaneous presence of the voltage and current during transition between the ON and OFF states, causing energy dissipation. The switching loss is one of the main losses in a high-frequency power conversion system [28,40]. The switching loss can be divided into turn-on ($E_{on}$) and turn-off losses ($E_{off}$). In addition, in a power conversion system, the dead time is the duration of time set to keep both the upper and lower switches from turning on simultaneously to avoid shoot-through. The losses during this period can be calculated as [10],

(14) $$E_{dead}=\int_{0}^{t_{d}}I_{dead}\times V_{dead}\;d\left(t\right),$$

where $t_{d}$ is the dead-time duration, and $V_{dead}$ and $I_{dead}$ are the voltage and current during the dead time, respectively. Therefore, the total loss of the system can be denoted as,

(15) $$P_{Total}=P_{Cond}+\left(E_{on}+E_{off}+E_{dead}\right)\times f_{sw}.$$

## 5. Numerical Modeling

**Figure 5:**
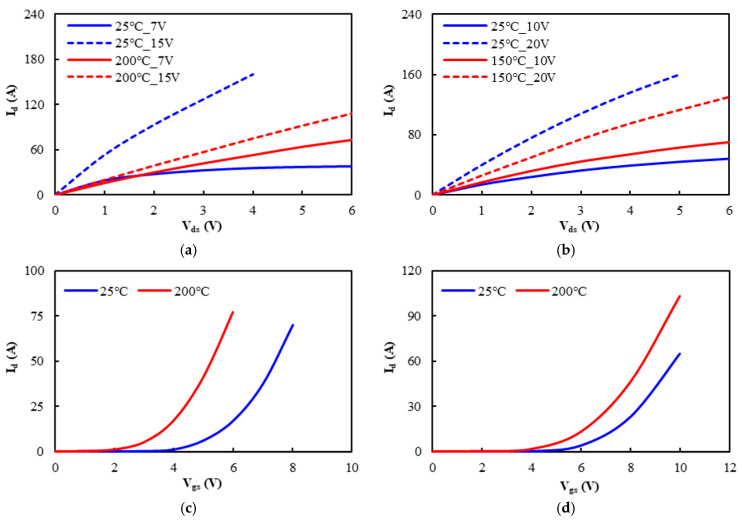

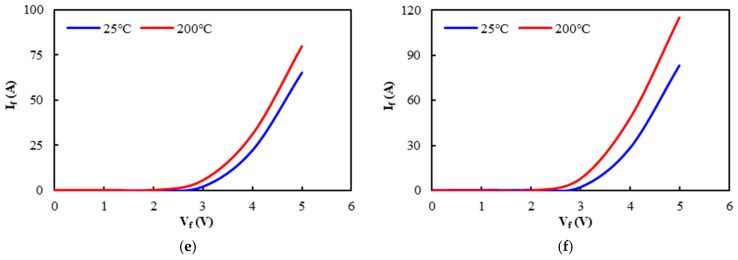
Output characteristics of the (**a**) SiC ITRI and (**b**) commercial power MOSFETs; transfer characteristics of the (**c**) SiC ITRI and (**d**) commercial power MOSFETs; body diode characteristics of the (**e**) SiC ITRI and (**f**) commercial power MOSFETs.

To accurately evaluate the system efficiency, power loss, and switching characteristics of the SiC MOSFET power switches of the totem-pole PFC boost converter during load operation, an ECCS model is created, which fully integrates an electromagnetic model, an equivalent circuit model, and an SiC MOSFET characterization model. Specifically, this model takes into account the parasitic effects on the switching transient behavior of the power switches. This three-dimensional (3D) quasi-static electromagnetic model using ANSYS^®^ Q3D (2022 R1) is utilized to extract the parasitic parameters of the current paths within the module [28,29,30], including parasitic inductance and parasitic capacitance, which are the key factors influencing the switching behavior. During the parasitic extraction using ANSYS^®^ Q3D, the finite element method (FEM) is performed to simulate the distribution of current within the module and the resulting electromagnetic effects. To raise the accuracy of the analysis, an adaptive mesh refinement technique is applied. By assigning the current sources and sinks to the internal current paths, parasitic parameters are extracted for each specific path, generating a parasitic parameter matrix that comprehensively depicts the self-inductance effect of the internal current and also the mutual inductance effect between different paths. The extracted parasitic parameter matrix combined with the equivalent circuit model of the boost converter is further used to simulate the switching behavior of the SiC MOSFET power switches during operation. This allows for a comprehensive consideration of the impact of parasitic effects on switching transients and power losses in numerical simulations. The proposed equivalent circuit model is based on the key characteristic curves provided in the component datasheet, including the output, transfer, and body diode characteristic curves, as shown in Figure 5. These characteristic curves are essential for precisely representing the power MOSFET devices’ switching performance and internal behaviors.

**Figure 6:**
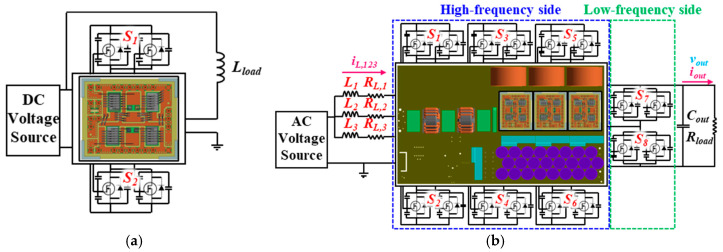
Proposed ECCS models for switching transient characterization and power loss estimation: (**a**) DPT; (**b**) interleaved totem-pole PFC boost converter.

During the DPT, only one power module is installed on the experimental power board for testing. Therefore, the DPT analysis model does not include the mutual inductance effects from the other two-phase circuits. The DPT analysis model, as shown in Figure 6a, is set up to match the actual experimental conditions. It includes an external 3 Ω gate resistor and a 154 µH load inductor. The environmental and initial conditions for this analysis are set to room temperature, a DC bus voltage of 1200 V, and a load current of 100 A. In contrast, the totem-pole PFC boost converter analysis model is more complex, as it includes a three-leg system consisting of three pairs of series-connected inductor and resistor, high-frequency side power switches, and low-frequency side power switches. In addition, the system also includes an output capacitor and a load resistor. The totem-pole PFC analysis model is shown in Figure 6b. The system’s AC power supply provides a single-phase AC input of 220 *V_rms_* with a frequency of 60 Hz. The high-frequency side switches operate at a switching frequency of 100 kHz and have a 120-degree phase shift, constituting a three-phase interleaved operation. To prevent simultaneous conduction of the upper-arm and lower-arm switches, a dead time of 400 ns is set, ensuring the stability and safety of the system’s operation. Noteworthy is that the control strategy of the totem-pole PFC system includes three main functions: voltage outer loop control, current inner loop control, and polarity detection. These functions work together to achieve efficient PF correction and stabilize the system’s DC output voltage. The main function of the voltage outer loop control is to stabilize the DC output voltage, allowing it to maintain at the set value even in the presence of load variations or input voltage fluctuations. This loop uses the feedback signal of the output voltage as input, compares it with the target voltage to generate an error signal, and then adjusts it through a proportional-integral (PI) controller. The output signal of the PI controller represents the reference value for the current inner loop, which is the target current amplitude on the AC side. This outer loop control mechanism allows the system to dynamically adjust the output voltage under different operating conditions so as to achieve a stable voltage output. The current inner loop control is responsible for tracking the target waveform of the AC-side current to ensure that it is in phase with the input voltage to satisfy the requirement for high PF operation. The inner loop control is based on the feedback signal of the input current. A comparator compares this signal with the target current provided by the outer loop, generating a current error signal. After the error signal is adjusted by the PI controller, it serves as the modulation reference for the pulse width modulation (PWM) control signal. PWM modulation is used for precise control of the high-frequency side switches to regulate the power flow such that the actual current strictly tracks the target waveform. The fast response characteristics of this current inner loop enable the system to effectively suppress current harmonics and so enhance power quality. The polarity detection function is a key technology in the totem-pole PFC system, primarily used to determine the changes in the polarity of the input AC voltage. The results of the polarity detection determine the conduction state of the low-frequency side SiC MOSFET power switches in order to warrant the correct power flow during both the positive and negative half cycles of the AC voltage. When the input voltage is in the positive half cycle, the positive polarity signal triggers the corresponding low-frequency side switch to turn on, allowing current to flow between the high-frequency side switches and the low-frequency side switch. During the negative half cycle, the other low-frequency side switch turns on to facilitate reverse power flow.

## 6. Results and Discussion

### 6.1. Characterization of DPT Switching Characteristics

**Figure 7:**
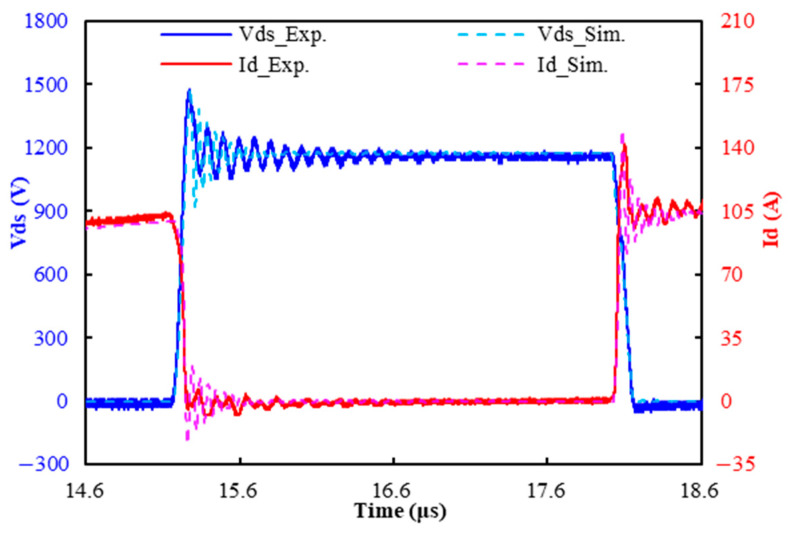
Modeled and measured voltage and current transient waveforms of the SiC MOSFET power module.

The waveforms obtained from the DPT experiment and the ECCS model are shown in Figure 7. From this figure, it can be observed that there is a good consistency between them, with only fair differences in the voltage and current oscillation frequencies. The oscillation frequency of the voltage and current is primarily influenced by the parasitic inductance and capacitance within the circuit. Therefore, the difference in oscillation frequencies between the simulation and experimental results can be attributed to the parasitic effects caused by the instruments, connection wires, and gate driver board. Table 2 lists the derived switching characteristics through the simulation and measurements, including the turn-on time ($t_{on}$), turn-off time ($t_{off}$), turn-on energy ($E_{on}$), turn-off energy ($E_{off}$), voltage spike, and current spike. The table reveals that there is a high degree of consistency in the calculated and measured switching characteristics, where the maximum difference is less than 6%, except for the turn-on energy, where the difference is as much as 16.5%. This is probably due to the addition of a filtering ferrite bead during the measurement to eliminate high-frequency noise. This creates certain flaws in the current waveform, and thus triggers the discrepancy.

### 6.2. Comparison of Interleaved and Non-Interleaved Totem-Pole PFC Boost Converters

**Figure 8:**
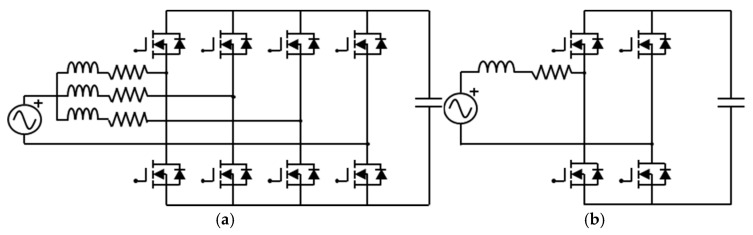
Schematics of circuit diagrams of (**a**) three-leg interleaved and (**b**) non-interleaved totem-pole PFC boost converter configurations.

In the totem-pole PFC boost converter, the three-leg interleaved and non-interleaved structures are the two most common circuit configurations, as shown in Figure 8. A comparative study of these two types of converters is carried out in terms of power loss and system efficiency. Table 3 lists the calculated power loss, system efficiency, and power factor for these two converter configurations using the proposed ECCS model. It is clearly found that the high-frequency side loss of the three-leg interleaved converter is much lower than that of the non-interleaved. This is mainly because, in the non-interleaved converter, all the current is carried by a single leg, which would lead to a higher current peak, further generating a greater conduction loss and switching loss. On the other hand, the interleaved converter evenly distributes the input current across these three legs through three-leg parallel operation, and this can reduce the current stress on an individual high-frequency switching device. Moreover, interleaving can offer the benefit of lowering current flowing through these switches. Conduction loss is proportional to the square of the current; consequently, with a smaller current amplitude, it can be significantly reduced. The operating frequency of the low-frequency side switching devices is the same as the AC grid frequency, which is 60 Hz. Compared to the high-frequency side, the switching frequency on the low-frequency side is rather low, and thus, the switching loss of the low-frequency side switches can be considered negligible. The main loss on the low-frequency side is conduction loss arising from the on-state resistance of the switching devices. Since the total current on the low-frequency side is essentially the same in both the interleaved and non-interleaved boost converters, and the number and characteristics of their low-frequency side switching devices are also identical, their conduction losses turn out to be very comparable. Due to the significant reduction in the high-frequency side losses and the three-leg interleaved operation that evenly distributes the input current load, the interleaved converter diminishes the current stress and switching loss on the switching devices. Consequently, the interleaved converter exhibits higher efficiency in high-frequency operation compared to the non-interleaved. Furthermore, because the three-leg interleaved operation staggers the current waveforms of each leg, the total input current ripple is repressed by the three-leg interleaving as a result of the ripple cancellation effect [38]. Hence, the input current waveform aligns more closely with a sine wave, thereby bringing about an improved power factor.

### 6.3. Closed-Loop Converter Operation and Power Loss Estimation

**Figure 9:**
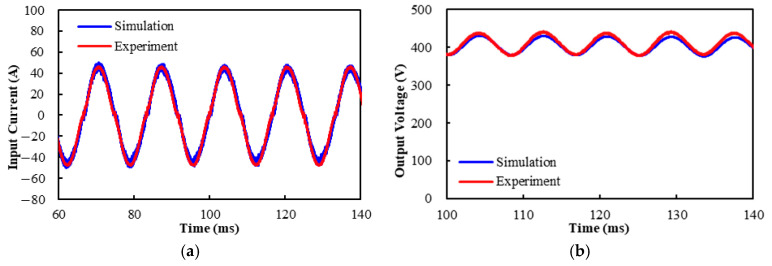
Measured and modeled input currents and output voltages: (**a**) Input current; (**b**) Output voltage.

To further validate the effectiveness of the ECCS model, a closed-loop system experiment is performed on the three-leg interleaved totem-pole PFC boost converter to characterize the input current, output voltage, and power loss. The simulation uses the same 220 *V_rms_*, 60 Hz AC voltage as the experiment, with a target DC voltage of 400 V and a rated power of 6.6 kW. Figure 9 presents the input current and output voltage obtained from both the experiment and simulation. It is evident to see that there is a slight difference in the calculated and measured waveform amplitudes, which can be stemmed from parasitic effects resulting from the measurement setup. Under room temperature conditions, the experiment demonstrates that the interleaved totem-pole PFC boost converter generates an output power of around 6.616 kW from an input power of about 6.888 kW, and the power efficiency is about 96.1%. In contrast, the ECCS exhibits that an output power of about 6.585 kW is taken from the input power of around 6.819 kW, suggesting a power efficiency of 96.6%. Clearly, the modeled power efficiency is very analogous to the experimental. Additionally, the peak input currents obtained from the experiment and simulation are 46.29 A and 47.86 A, respectively, with a difference of approximately 3.3%. On the other hand, the corresponding peak output voltages are 438.48 V and 425.01 V, with a difference of around 3.1%. Clearly, the simulation results agree well with the experimental.

The estimated conduction losses, switching losses, body diode losses and their sum of the high-frequency side (i.e., S1, S2, S3, S4, S5, and S6) and low-frequency side switches (i.e., S7 and S8) in the three-leg interleaved totem-pole PFC boost converter, are listed in Table 4, along with their total loss. It is important to note that each of these power switches consists of two SiC MOSFET devices, and the data listed in the table are the average power loss value over one cycle. Noticeably, as can be seen in the table, the total power loss of each of these six high-frequency side switches is very comparable, with an average value of about 18.90 W. The ratios of the conduction loss, switching loss, and body diode loss of these power switches to their corresponding total power loss are 7.3%, 43.2%, and 49.4%. It is observed that the body diode loss dominates the total power loss of these power switches, followed by the switching loss and the conduction loss. In other words, the conduction loss takes only the least portion of the total power loss, which can be ascribed to the lower on-state resistance of the SiC MOSFET devices [41]. In contrast, the dominance of the body diode loss can be a result of the high-frequency switching, leading to a larger reverse recovery loss.

The power losses of the S1 high-frequency side switch, S7 low-frequency side switch and one power inductor as a function of temperature are listed in Table 5, where the temperature varies from 25 °C to 175 °C, with an increment of 75 °C. It shows that the low-frequency side losses increase with the temperature because of the increased on-state resistance of the power MOSFET devices. Similar results can be also found in the loss of the high-frequency side switches and power inductors. It is important to note that, because the forward voltage of the body diode has a negative temperature coefficient, the body diode loss decreases as temperature increases [29,30]. This counteracting effect is the main reason for the unimportant temperature dependence of the loss of the high-frequency side switch.

### 6.4. Performance Comparison Between SiC MOSFET-Based and Si IGBT-Based Interleaved PFC Boost Converters

The power loss and system efficiency of the three-leg interleaved totem-pole PFC boost converter, using the SiC MOSFET and Si IGBT devices, are compared through the proposed ECCS model, and the calculated results are displayed in Table 6. It is worth noting that the low-frequency side switches in these two types of boost converters use the same commercial SiC MOSFET devices. The table evidently reveals that the SiC MOSFET-based PFC boost converter is much superior to the Si IGBT-based one, in terms of power loss and efficiency. On the contrary, since the same switching devices on the low-frequency side are used, their power losses are nearly identical. More specifically, the total loss of the Si IGBT-based PFC boost converter is 283.07 W, while that of the SiC MOSFET-based one is only 164.48 W. The higher total loss of the Si IGBT-based PFC boost converter is mainly due to the inherent switching characteristics of these Si IGBT devices, in which they possess a slower switching speed compared to the SiC MOSFET devices, making them inclined to an increased switching loss in this high-frequency system. Additionally, Si IGBT devices lack an intrinsic body diode and thus require external Si diodes to carry reverse currents, having poorer reverse recovery characteristics and leading to a greater loss. These two factors contribute to a much more significant power loss of the Si IGBT devices. To mitigate the loss and improve efficiency, Schottky diodes can be used as external diodes due to their superior reverse recovery characteristics. However, this would inevitably come with the trade-off of increased costs. The significant increase in loss of the high-frequency side switches is the main cause for the difference in the total loss between them. Due to the increase in power loss, the system efficiency drops from 96.64% for the SiC MOSFET-based PFC boost converter to 92.43% for the Si IGBT-based one. The results suggest that, despite higher cost, the SiC MOSFET devices are particularly advantageous for the high-frequency, high-efficiency PFC boost converter applications because of their fast-switching characteristics, low switching losses, and excellent body diode performance.

### 6.5. Parametric Analysis

Power loss is a critical factor affecting device junction temperature and eventually the lifetime of the power module. A design guideline for minimizing power loss so as to enhance system efficiency, thermal performance, and lifetime of the developed PFC boost converter is sought through parametric analysis using the validated ECCS model. In this parametric study, the effect of four operation parameters on the power losses of the high-frequency (S1~S6) and the low-frequency side switches (S7~S8), and their sum is assessed. The operation parameters considered are switching frequency, DC output voltage, and the inductance of the power inductors. It is particular noteworthy that throughout the parametric study, the output power is maintained at a constant 6.6 kW. Additionally, the power inductor and output capacitor are also influenced by changes in these parameters, but their impacts are not considered in this parametric study.

**Figure 10:**
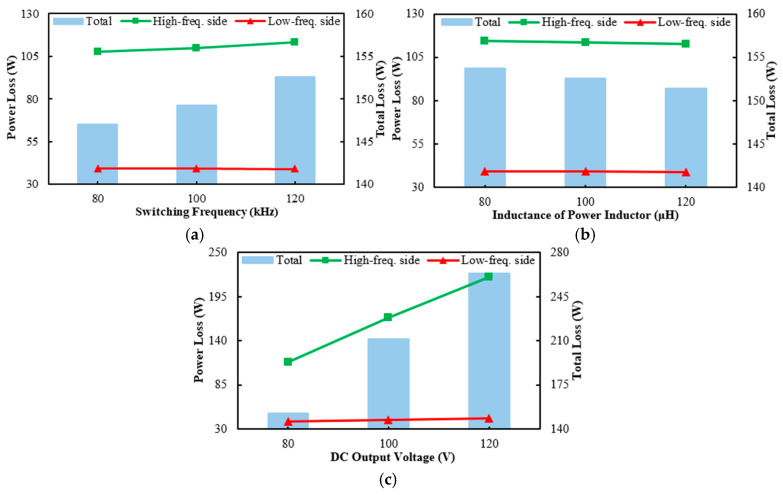
Effect of four operational parameters on the power losses of the three-leg interleaved totem-pole PFC boost converter: (**a**) ambient temperature, (**b**) inductance of power inductor, and (**c**) DC output voltage.

The effect of switching frequency on power losses is assessed, where the switching frequency is varied from 60 kHz to 100 kHz in 20 kHz increment, and the results are illustrated in Figure 10a. Noteworthy is that the power inductor remains unchanged throughout this assessment, ensuring that the observed variations in power losses are solely attributed to the switching frequency. The figure exhibits that the power loss of the high-frequency side switches increases with the switching frequency. This is mainly due to the increased number of switching operations within each cycle, leading to a higher switching loss. Moreover, a higher switching frequency amplifies the parasitic effects, thus bringing about a higher voltage spike and ringing, which further increase the conduction loss and body diode loss. On the other hand, the low-frequency side switches primarily operate at line frequency, so changes in the switching frequency have a minimal impact on their loss. While a higher switching frequency tends to lead to an increased loss, it would also reduce the current ripple, enabling the use of a smaller power inductor. This reduction in the size of the inductors helps to achieve a more compact overall design.

The dependence of the power losses on the inductance of the power inductors is presented in Figure 10b, where the inductance varies from 220 μH to 320 μH with an increment of 50 μH. The figure demonstrates that the losses of both the high-frequency and low-frequency side switches decrease marginally with the inductance of the power inductors, with the main reason being that an increased power inductor’s inductance tends to diminish the amplitude of the input current ripple, which smooths the instantaneous current through the SiC power MOSFETs. This would thus reduce the switching and conduction losses of the high-frequency side switches and the conduction loss of the low-frequency side switches, thereby leading to a decreased total loss. In addition, a larger inductance can reduce the total harmonic distortion and so improve the power factor. It is, however, important to note that a greater inductance value implies a need for a larger inductor, which would unfavorably increase the system’s size and weight. Additionally, a larger inductor suggests an extended current regulation time of the system, potentially affecting the dynamic response.

Figure 10c displays the influence of the DC output voltage on power losses, where three DC output voltages, namely 400 V, 500 V, and 600 V, are considered. It is essential to note that in order to maintain a constant output power of 6.6 kW, the load resistance changes from 24.24 Ω to 37.88 Ω and to 54.55 Ω, respectively, as the output voltage increases from 400 V to 500 V and to 600 V. Apparently, an increasing output voltage would lead to a dramatical rise in the total loss of the power switches on the high-frequency side. This is largely due to the growth in the switching loss with an increasing output voltage. In contrast, a slight increase in the power loss of the low-frequency side switches with an increasing output voltage is observed. This can be principally explained by the fact that an increase in the output voltage leads to a rise in the current ripple, which in turn causes a higher conduction loss.

## 7. Conclusions

This study carries out an extensive characterization of the dynamic behavior of the developed 6.6 kW bidirectional bridgeless three-leg interleaved totem-pole PFC boost converter for the front-end stage of electric vehicle (EV) onboard charger (OBC) applications during load cycles using an ECCS model. This ECCS model integrates the electromagnetic model, equivalent circuit model, and SiC MOSFET characterization mode, where the parasitic effect within the converter system on the dynamic characteristics of the PFC boost converter is included. Experimental validation through the DPT and closed-loop converter operation is performed on the proposed ECCS model. Besides, the performance of the proposed interleaved PFC boost converter is compared to that of the non-interleaved, and that of the SiC MOSFET-based PFC boost converter is contrasted with that of the Si IGBT-based one. Finally, the effect of four operation parameters on the performance of the proposed 6.6 kW PFC boost converter is addressed. In conclusion, a number of essential remarks are drawn in the following:

1. The simulated switching characteristics and the voltage and current waveforms under closed-loop operation match very well with the experimental results, proving the effectiveness of the proposed ECCS model.
2. The three-leg interleaved design offers reduced power loss and improved efficiency compared to the non-interleaved, primarily attributed to the even distribution of the total input current across these three legs. Additionally, the three-leg interleaving achieves a reduced total input current ripple due to the ripple cancellation effect, leading to an improvement in the power factor.
3. In the interleaved totem-pole PFC, the power loss of the high-frequency side switches is appreciably higher than those of the low-frequency side switches. This is because the low-frequency side loss is mostly the conduction loss of the power switches, with the switching loss and body diode loss being almost negligible. In contrast, the high-frequency side loss is dominated by the switching loss and the body diode loss of the power switches, arising from the high-frequency switching, potentially leading to a larger reverse recovery loss. Additionally, the conduction loss takes a very small part of the high-frequency side loss during the closed-loop converter operation. This can be attributed to a lower current flowing through these switches and a lower on-state resistance of the SiC MOSFET devices.
4. The conduction loss of the high-frequency side switches increases with the temperature due to the corresponding rise in the on-state resistance. Conversely, the body diode loss decreases with the temperature, because of the negative temperature coefficient of the body diodes. This result offsets the increase in the conduction loss with the temperature, causing a trivial temperature dependence of the high-frequency side loss.
5. The performance comparison between the SiC MOSFET-based and the Si IGBT-based PFC boost converter highlights the clear advantages of the SiC MOSFET devices for high-frequency, high-efficiency applications owing to their faster switching speed, lower switching loss, and better body diode performance.
6. The parametric analysis shows that increasing the line frequency and power inductor, as well as decreasing the output voltage, can reduce high-frequency side losses. Low-frequency side losses can be reduced by lowering the temperature, and increasing the line frequency and power inductor. Among these, the output voltage has the most significant impact on the Totem-Pole PFC converter losses.

## Figures and Tables

**Table 1:** Interleaved totem-pole PFC converter parameters.

$P_{out}$ (kW)	$V_{out}$ (V)	$\Delta i_{L,123}$ (%)	$\Delta V_{out}$ (%)	$f_{sw}$ (kHz)	$f_{line}$ (Hz)
6.6	400	5	635	100	60
$C_{out}$ (μF)	$L_{123}$ (μH)	$R_{L,123}$ (mΩ)	$R_{Gate}$ (Ω)	$R_{out}$ (Ω)	$t_{Dead}$ (ns)
900	270	38	3	24.2	400

**Table 2:** Comparison of DPT switching characteristics between simulation and experiment.

	$t_{on}$	$t_{off}$	$E_{on}$	$E_{off}$	$V_{spike}$	$I_{spike}$
Experiment	158 ns	250 ns	6.4 mJ	2.4 mJ	1476 V	142 A
Simulation	164 ns	247 ns	5.3 mJ	2.3 mJ	1480 V	150 A
Difference	3.6%	1.2%	16.5%	4.0%	0.3%	5.6%

**Table 3:** Performance comparison of three-leg interleaved and non-interleaved totem-pole PFC boost converters.

	Three-Leg Interleaved Design	Non-Interleaved Design
High-frequency side loss (W)	113.41	144.91
Low-frequency side loss (W)	39.17	40.25
Power inductor loss (W)	11.90	12.48
Total power loss (W)	164.48	197.64
Efficiency (%)	96.64	94.96
Power factor	0.996	0.987

**Table 4:** Power losses of three-leg interleaved totem-pole PFC boost converter.

Switch	Conduction (W)	Switching (W)	Body Diode (W)	Sum (W)	Total (W)
S1	1.386	8.178	9.354	18.918	164.477
S2	1.393	8.186	9.298	18.877	
S3	1.387	8.171	9.346	18.903	
S4	1.392	8.188	9.344	18.924	
S5	1.386	8.158	9.322	18.866	
S6	1.392	8.191	9.342	18.926	
S7	19.557	-	-	19.557	
S8	19.609	-	-	19.609	
	per leg				
Inductor	3.966	-	-	11.898	

**Table 5:** Temperature-dependent power losses of S1 high-frequency side switch, S7 low-frequency side switch and one power inductor. (unit: W).

Temp.(°C)	High-Freq. Side Switch (W)			Sum (W)	Low-Freq. Side Switch (W)	Inductor (W)
	Conduction	Switching	Body Diode			
25	1.386	8.178	9.354	18.918	19.557	3.966
100	1.796	8.176	9.001	18.973	25.347	5.140
175	2.233	8.175	8.618	19.025	31.154	6.318

**Table 6:** Performance comparison of the SiC MOSFET-based and Si IGBT-based three-leg interleaved totem-pole PFC boost converter.

	SiC MOSFET-Based	Si IGBT-Based
High-frequency side loss (W)	113.41	230.55
Low-frequency side loss (W)	39.17	40.28
Power inductor loss (W)	11.90	12.24
Total power loss (W)	164.48	283.07
Efficiency (%)	96.64	92.43
Power factor	0.996	0.990

## Data Availability

The original contributions presented in the study are included in the article, further inquiries can be directed to the corresponding author.
